# Modeling relaxed policies for discontinuation of methicillin-resistant *Staphylococcus aureus* contact precautions

**DOI:** 10.1017/ice.2024.23

**Published:** 2024-07

**Authors:** Jiaming Cui, Jack Heavey, Leo Lin, Eili Y. Klein, Gregory R. Madden, Costi D. Sifri, Bryan Lewis, Anil K. Vullikanti, B. Aditya Prakash

**Affiliations:** 1 College of Computing, Georgia Institute of Technology, Atlanta, Georgia; 2 Department of Computer Science, University of Virginia, Charlottesville, Virginia; 3 Center for Disease Dynamics, Economics & Policy, Washington, DC; 4 Department of Emergency Medicine, Johns Hopkins School of Medicine, Baltimore, Maryland; 5 Department of Epidemiology, Johns Hopkins Bloomberg School of Public Health, Baltimore, Maryland; 6 Division of Infectious Diseases & International Health, Department of Medicine, University of Virginia School of Medicine, Charlottesville, Virginia; 7 Office of Hospital Epidemiology/Infection Prevention & Control, UVA Health, Charlottesville, Virginia; 8 Biocomplexity Institute, University of Virginia, Charlottesville, Virginia

## Abstract

**Objective:**

To evaluate the economic costs of reducing the University of Virginia Hospital’s present “3-negative” policy, which continues methicillin-resistant *Staphylococcus aureus* (MRSA) contact precautions until patients receive 3 consecutive negative test results, to either 2 or 1 negative.

**Design:**

Cost-effective analysis.

**Settings:**

The University of Virginia Hospital.

**Patients:**

The study included data from 41,216 patients from 2015 to 2019.

**Methods:**

We developed a model for MRSA transmission in the University of Virginia Hospital, accounting for both environmental contamination and interactions between patients and providers, which were derived from electronic health record (EHR) data. The model was fit to MRSA incidence over the study period under the current 3-negative clearance policy. A counterfactual simulation was used to estimate outcomes and costs for 2- and 1-negative policies compared with the current 3-negative policy.

**Results:**

Our findings suggest that 2-negative and 1-negative policies would have led to 6 (95% CI, −30 to 44; *P* < .001) and 17 (95% CI, −23 to 59; −10.1% to 25.8%; *P* < .001) more MRSA cases, respectively, at the hospital over the study period. Overall, the 1-negative policy has statistically significantly lower costs ($628,452; 95% CI, $513,592–$752,148) annually (*P* < .001) in US dollars, inflation-adjusted for 2023) than the 2-negative policy ($687,946; 95% CI, $562,522–$812,662) and 3-negative ($702,823; 95% CI, $577,277–$846,605).

**Conclusions:**

A single negative MRSA nares PCR test may provide sufficient evidence to discontinue MRSA contact precautions, and it may be the most cost-effective option.

Methicillin-resistant *Staphylococcus aureus* (MRSA) is a leading source of healthcare-associated infections (HAIs) in the United States. In hospitals, contact precautions are typically applied to patients with known MRSA status or who test positive on screening tests to control in-hospital transmission.^1–3^ Nares polymerase chain reaction (PCR) tests are recommended for guiding decisions on discontinuing MRSA precautions,^4^ also known as releasing policies. Although 1–3 consecutive negative test results from the nares are common thresholds for discontinuation of contact precautions, these releasing policies are often arbitrary, lacking substantial evidence and neglecting patient-specific factors. For instance, the University of Virginia (UVA) Hospital employs a “3-negative policy” under which contact precautions are discontinued after 3 consecutive negative test results. However, whether this policy is optimal in terms of balancing costs and controlling MRSA transmission is unknown.^5^

If MRSA surveillance testing were always accurate, then a single-negative policy would be optimal. However, several factors, including sensitivity of the test, low bacterial load at time of collection, and sampling error, abrogate the accuracy of testing. Repeated testing improves the sensitivity and thus can extend precautions in patients that may otherwise be assumed clear. However, requiring gowns and gloves and other resources to isolate MRSA patients is expensive, with an average estimated cost of >$400 per day per patient.^6^ In addition, although the extent of side-effects of contact precautions remains uncertain,^7,8^ there are potential harms due to extended contact precautions,^4,9^ including reductions in provider visits to patient rooms,^10^ which can increase the risk of depression.^7^ Thus, releasing policies must balance the potential harms from contact precautions against the potential prevention of transmission.

Few systematic analyses have evaluated the optimal length of releasing policies. Prior research on evaluating releasing policies^11,12^ have focused on individual-level risk estimation using electronic health record (EHR) data. We examined the trade-off between increased transmission (which would raise treatment costs) and reduced costs from fewer precaution days at the hospital level. We used a mathematical model to simulate outcomes because no empirical data exist.

## Methods

### 2-Mode–Precaution model

**Figure 1.**
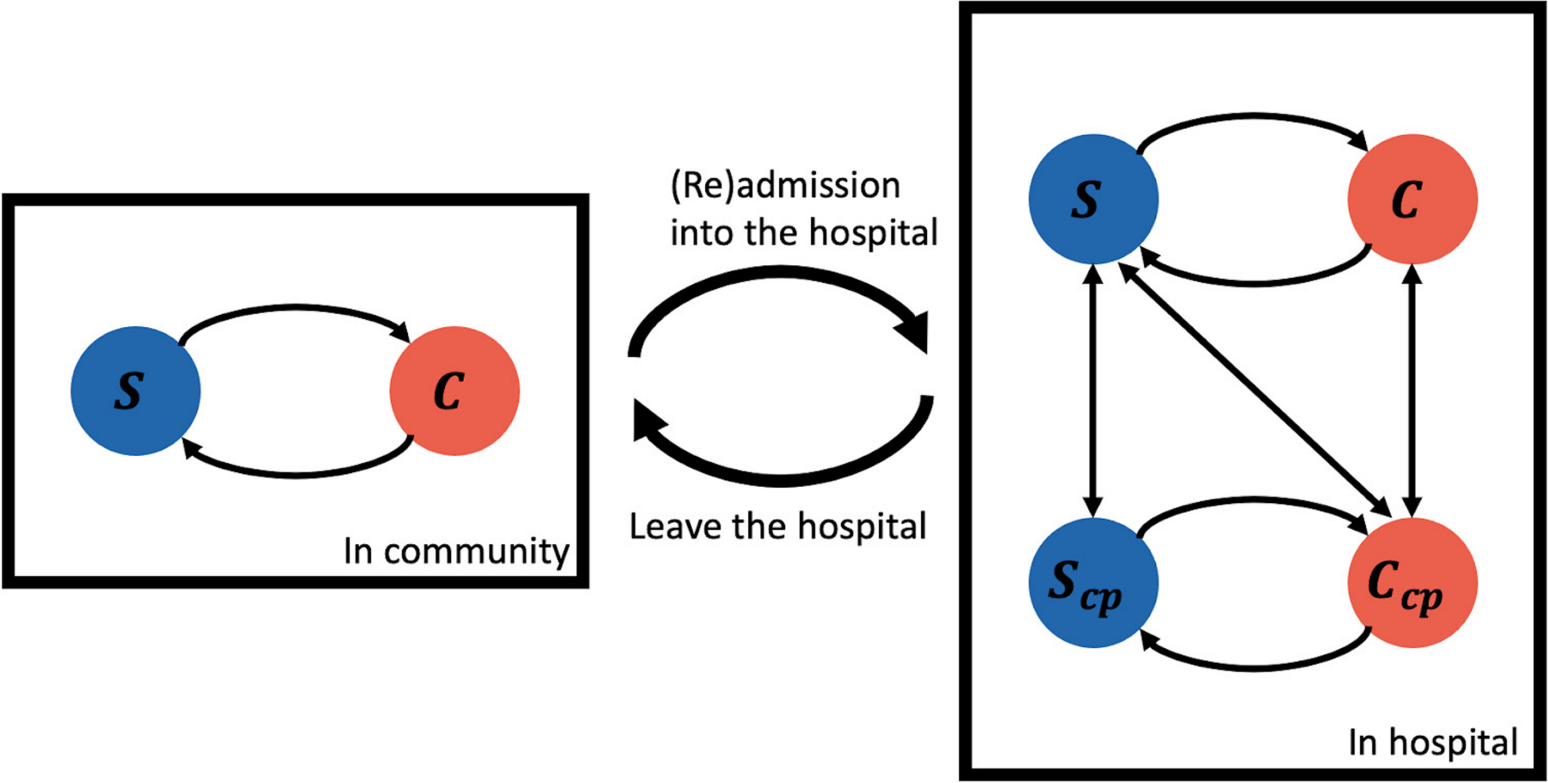
The diagram of states for patients in the 2-Mode–Precaution model. There are 6 states in the 2-Mode-Precaution model: four for patients in the hospital (ie, 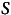 , susceptible, out of contact precaution, 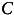 , carriage, out of contact precaution, 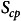 , susceptible, under contact precaution, and 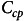 , carriage, under contact precaution) and 2 ( 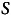 and 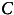 ) are for patients outside the hospital, or in the community. Each patient, HCW, and location is associated with a pathogen load, which is transferred through contacts between people and locations. A patient is infected with a probability that depends on the load they have accumulated. Load on all entities decays at a steady rate.

In this study, we propose a new dynamical system model, referred to as the 2-Mode–Precaution model, to represent MRSA transmission pathways in the University of Virginia Hospital. This model extends a prior model, referred to as the 2-Mode–Susceptible-Infectious-Susceptible (SIS) epidemic model,^13,14^ which has been proposed for capturing MRSA transmission dynamics but does not account for contact precautions. Our model includes 3 types of entities: patients, healthcare workers (HCWs), and locations. A key aspect of the model is the contact network, which specifies the contacts between patients and HCWs and the locations where these happen. This information is not directly available and is inferred from EHR data through tables (eg, medicine administration). Each patient in the hospital is in one of the following states: 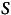 (susceptible), 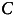 (carriage), 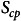 (susceptible, under contact precaution) and 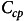 (carriage, under contact precaution). Patients outside the hospital can be either in 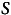 or 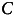 states. Figure 1 shows the state transition diagram of the 2-mode–precaution model. HCWs and locations are only associated with pathogen load (so they can be considered as being in the carriage state).

Following the approach of previous research,^13^ the 2-Mode–Precaution model has 3 parts: (1) transfer of pathogen load between entities, (2) patient infection (based on their pathogen load), and (3) decay in load. We assumed that a contact between entities 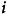 and 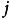 results in transfer of pathogen load between them and averages the load between them. The entire set of pathogen-transfer events can be captured by application of a pathogen-transfer matrix constructed from the network. A patient moves from a susceptible state ( 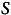 or 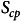 ) to a carriage state ( 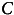 or 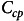 ) with a probability determined by a dose–response function. This probability increases with the patient’s pathogen load. Patients in the carriage state are assumed to shed at a higher rate. The shedding continues until the patient recovers.

A patient in state 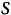 or 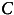 moves to state 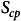 or 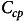 respectively, if they are in the hospital, and are put under contact precautions. For every patient under contact precaution (ie, in 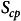 and 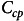 ), contact precautions reduce the pathogen load transferred via contacts by an effectiveness factor 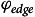 (see Supplementary Material online for more details). Notably, the University of Virginia Hospital has no general policy for testing. Instead, the tests are performed based on the clinician’s request. Therefore, in this work, we assumed that the testing rates and parameters in the precaution model were captured in the transition probabilities between different states, which were calibrated from MRSA infection data. For instance, 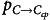 represents the transition probability from state 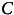 to state 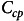 , corresponding to patients who tested positive for MRSA and were placed under contact precautions. Similarly, 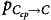 denotes the transition probability from state 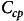 to state 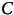 , corresponding to patients who were wrongly released due to false-negative MRSA tests. Such assumptions are reasonable because the carriage state in the 2-Mode–SIS model can represent both MRSA-colonized and -infected patients. Undetected colonized MRSA cases may have existed due to undertesting, false negatives, and/or MRSA infections occurring after hospital discharge. We also included parameters to account for importation of cases, following existing research.^15,16^ More details on the 2-Mode–Precaution model are provided in the Supplementary Material (online).

### Parameter adjustment method

The most clinically significant parameter changes in the 2-Mode–Precaution model for estimating outcomes for different releasing polices are 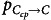 (the transition probability from 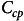 to 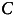 ) and 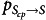 (transition probability from 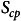 to 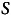 ). We denote the different policies as 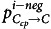 , where *i* is the number of tests in the policy. Other non–precaution-based infection controls and prevention measures are assumed to remain constant (eg, terminal room disinfection, standard hand hygiene practices, antimicrobial use). For 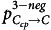 , only patients with 3 consecutive negative tests were released. Under the 2-negative policy ( 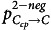 ), patients with the first 2 tests negative were released without a third test. For 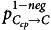 , patients under contact precautions were released after a negative test. We used 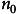 and 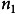 to represent the number of records with 3 consecutive negative tests (ie, negative–negative–negative) and 2 negative test results followed by 1 positive result (ie, negative–negative–positive) in the EHR data set. With the 2-negative policy, 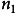 more patients would be released. Hence, we have 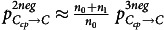 . Similarly, under the 3-negative policy, patients must wait for 3 consecutive negative tests to be released. However, under the 2-negative policy, patients only needed to wait for 2 consecutive negative tests, which indicated a shorter waiting time and hence a larger transfer probability. We used 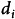 to capture the average number of days to obtain *i* number of consecutive negative tests after the initial positive test. By assuming that the average days follow a geometric distribution parameterized by 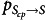 , then 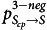 and 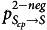 should be proportional to 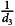 and 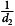 . Therefore, we have 
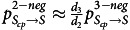
 . Similarly, we have 
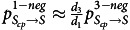
 . The exact value for 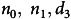 , and 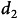 are listed in the Supplementary Material (online).

### Costs for different policies

To quantitatively examine the tradeoff between the number of additional MRSA cases and the number of precaution days saved under the relaxed policies, we calculated the total cost for different policies. We used costs reported in a retrospective study in a Canadian hospital from 2005 to 2010,^6^ which provided the estimated treatment (eg, laboratory cost, infection control time, housekeeping) and precaution (eg, private room and length of stay cost per day) cost for colonized patients and infected patients (values were adjusted to 2010 Canadian dollars in that study). As the study mentions, 88.9% of cases were colonized.^17^ The treatment costs (which are different for these 2 patient groups) are derived from multiplying the number of MRSA patients by the treatment cost per patient. Finally, we converted their values to 2010 US dollars,^18^ then we adjusted for inflation to 2023,^19^ for a final estimated cost of treatment of $435.89 in 2023 for MRSA-colonized patients and $459.13 in 2023 for MRSA-infected patients.

The cost of precautions was similarly calculated by multiplying the number of precaution days (the total number of patients under precaution each day) by the precaution cost estimates from the literature.^6^ After conversion, the estimated cost of precaution is $403.58 in 2023 for colonized patients and $2,110.90 in 2023 for infected patients. We focused only on the economic costs incurred by the University of Virginia Hospital. Patient costs, the downstream influence, and costs to the community or other healthcare facilities were not included.

### Data set

We constructed heterogeneous contact networks using EHR data collected at the University of Virginia Hospital. These records combine inpatient data, doctor’s notes, and medication administration data, which document the location and time of interactions between patients and healthcare workers (HCWs). We then aggregated this information into daily networks, connecting 2 nodes (patients, HCWs, and locations) in the contact networks if they have at least 1 contact on a given day. From January 1, 2015, to December 31, 2019, the constructed networks included 41,216 patients, 14,392 healthcare workers, and 685 locations across all departments within the hospital. The weekly number of incident MRSA cases was obtained using both MRSA-positive cultures and PCR nares surveillance swabs. For removal of precautions, we only considered negative PCR results because negative culture tests are not used to remove individuals from contact precautions at the University of Virginia Hospital. From January 1, 2015, to December 31, 2019, 22,825 PCR tests were performed on 15,806 patients based on clinician orders. These tests resulted in 2,481 positive and 20,344 negative outcomes, with 1,800 unique patients having at least 1 positive test. Although not all patients in the hospital were tested, potential undetected or colonized patients are accounted for in the carriage state of our 2-Mode–Precaution model. Further details are available in the Supplementary Material (online).

### Calibration and setup

As described in the introduction section, the University of Virginia Hospital employs a “3-negative policy” under which contact precautions are discontinued after 3 consecutive negative test results. Therefore, to infer the parameters for the 3-negative policy, we used the ensemble adjustment Kalman filter (EAKF),^20^ which has been widely used for epidemiological models of various healthcare-associated infections, including MRSA, and has demonstrated good performance.^16,21,22^ Specifically, we calibrated the number of cases transferred from 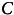 to 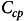 (ie, patients who were tested based on clinician orders and turned out to be positive) to the incident MRSA case numbers. Calibration was restricted to known incident hospital MRSA cases because incident cases outside the hospital are not recorded as part of the EHR data. The effectiveness of the calibration procedure on both synthetic data and real-world data is demonstrated in the Supplementary Material (online).

We used the parameter adjustment method to estimate the difference of outcomes between the current 3-negative policy and the potential 2- and 1-negative policies. We ran each simulation 300 times using these adjusted parameters to estimate outcomes, and we calculated the costs associated with each policy to compare their effectiveness.

## Results

### Number of detected MRSA cases

**Figure 2.**
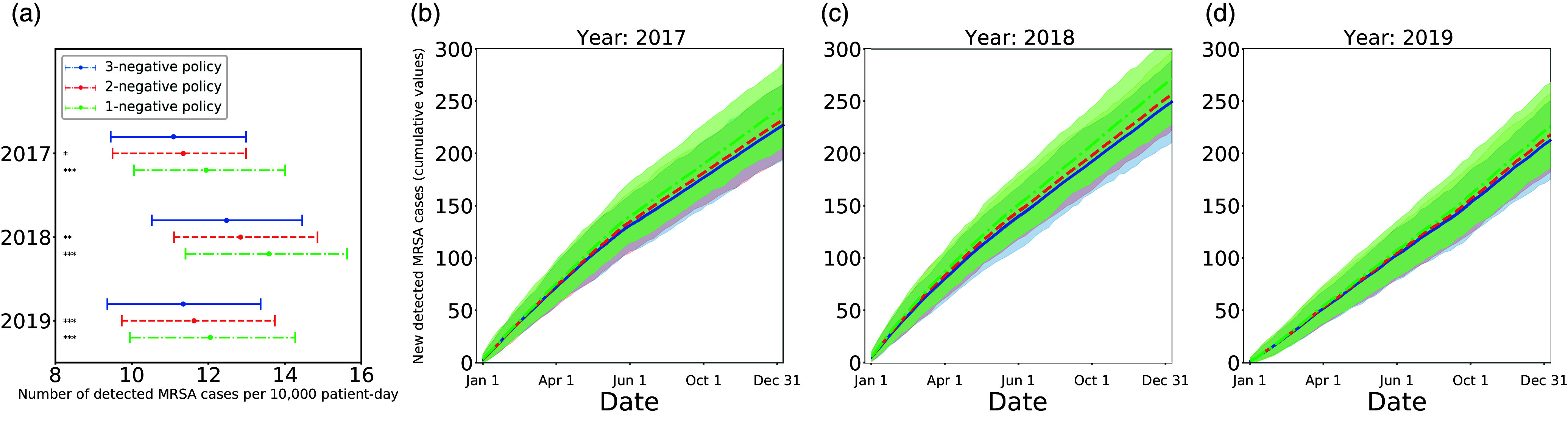
The 2-negative and 1-negative policies led to more new detected MRSA cases compared to the current 3-negative policy. (a) Blue dots and error bars indicate the mean values and 95% confidence intervals for detected MRSA cases per 10,000 patient days under the 3-negative policy, as determined by calibration. The red and green dots and error bars represent the estimated mean values and 95% confidence intervals for the number of detected MRSA cases per 10,000 patient days under the 2-negative and 1-negative policies, respectively. To demonstrate the differences between the 3-negative policy and the 2-negative and 1-negative policies, we employed the 2-sample *t* test (*P < .05; **P < .01; ***P < .001). The x-axis is the number of detected MRSA cases per 10,000 patient days, and the y-axis corresponds to 2017, 2018, and 2019. (b–d) The blue curves and shaded regions represent the mean value and 95% confidence interval for the cumulative number of detected MRSA cases under the 3-negative policy as determined by calibration. The red and green curves and shaded areas represent the estimated number of detected MRSA cases for 2-negative and 1-negative policies, respectively. The x-axis is the date, and the y-axis is the cumulative value for detected MRSA cases. (a) 2017. (b) 2018. (c) 2019.

On average, the 2-negative policy led to 2.56% more MRSA cases (95% confidence interval [CI], −13.2% to 19.1%; *P* < .001) than the 3-negative policy, although the 1-negative policy resulted in 7.59% more MRSA cases (95% CI, −10.1% to 25.8%; *P* < .001) during 2017–2019 (Fig. 2). Despite the large 95% CI owing to the inherent high variance in agent-based model simulations, the statistical 2-sample *t* tests revealed that both the 2-negative and 1-negative policies would lead to an increase in the number of detected MRSA cases per 10,000 patient days, compared to the 3-negative policy. Additional results can be found in the Supplementary Material (online).

**Figure 3.**
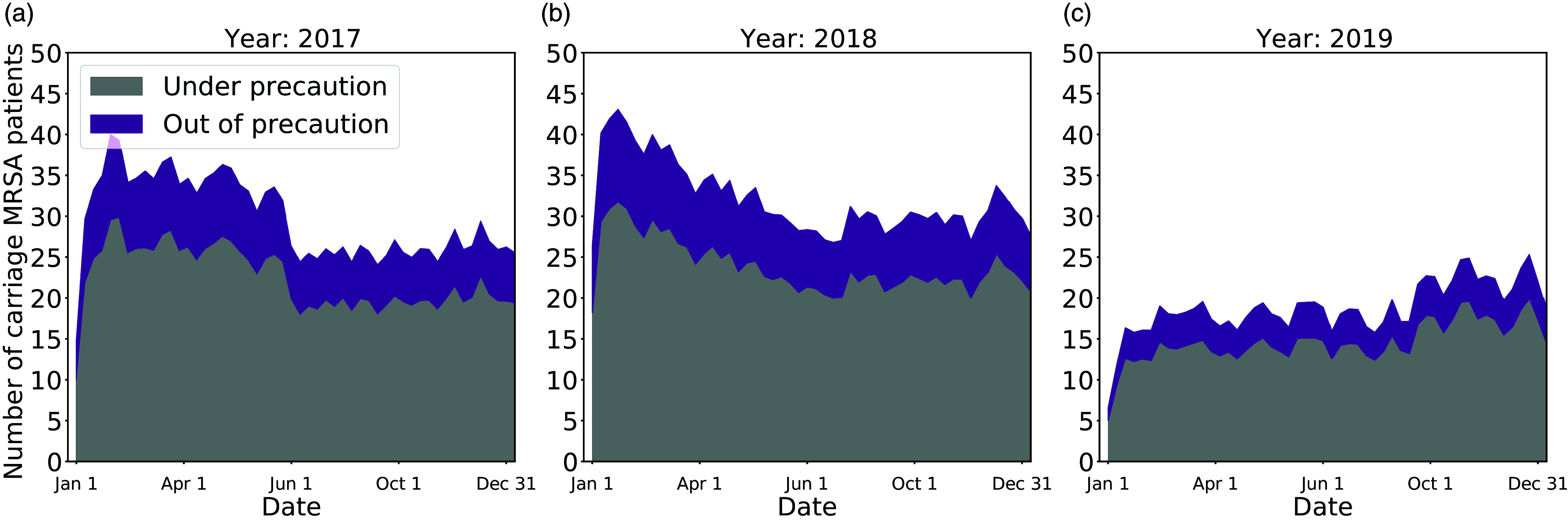
Most MRSA cases are under contact precautions. The shaded areas represent the weekly number of in-hospital patients in 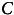 and 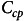 states under the 1-negative policy. The gray and purple sections correspond to cases that are under or not under contact precautions, respectively. (a) 2017. (b) 2018. (c) 2019.

Although removing contact precautions earlier did lead to more cases, the false-negative rate was low, so most MRSA cases remained under contact precautions in the hospital, even under the 1-negative policy (Fig. 3). Under the 1-negative policy, 75.77% (95% CI, 74.23%–77.05%) of MRSA cases remained under contact precautions.

### Shorter contact precaution durations for 2-negative and 1-negative policies

**Figure 4.**
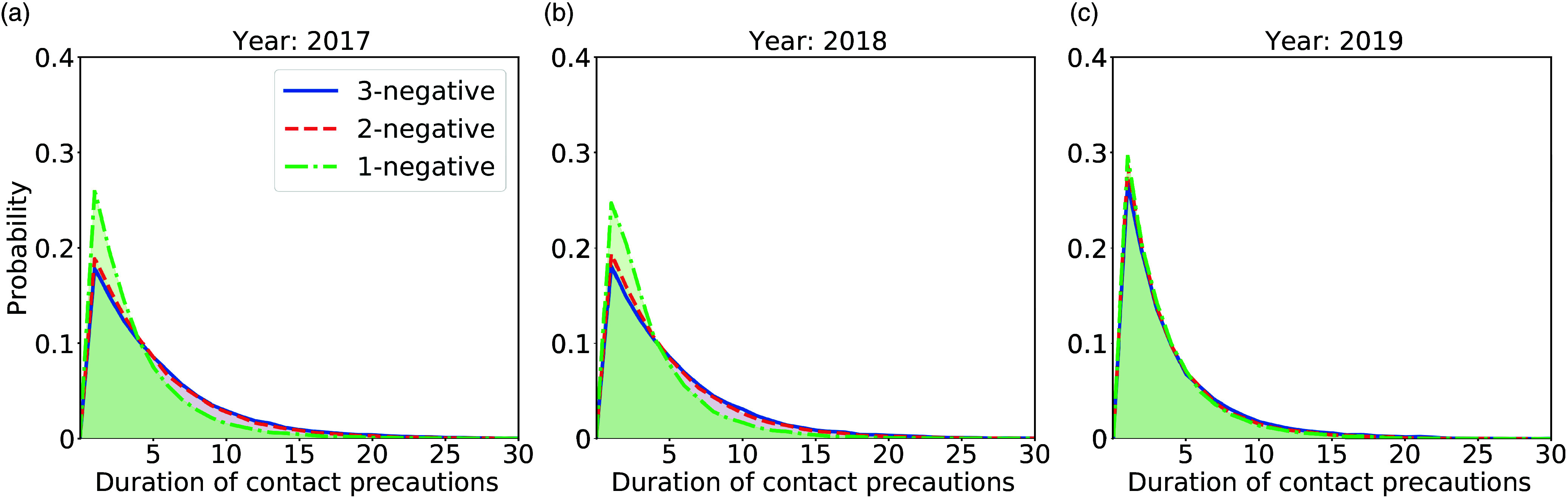
Distribution for contact precaution durations related to MRSA. The blue, red, and green curves represent the distribution for 3-, 2-, and 1-negative policies, respectively. The x-axis is the contact precaution durations in days, and the y-axis is the probability. (a) 2017. (b) 2018. (c) 2019.

The average contact precaution durations over 2017–2019 for the 2- and 1-negative policies were 5.56 days (SD, 4.05), and 4.75 days (SD, 3.35), respectively. These were both shorter than the 3-negative policy at 5.88 days (SD, 4.39) (Fig. 4). The shorter durations led to lower costs for precautions (Table 1). For the 2-negative policy, the estimated total cost, $687,946 (95% CI, $562,522–$812,662), was marginally lower than the 3-negative policy, $702,823 (95% CI, $577,277–$846,605) with *P* < .005 for the 2-sample *t* test). Meanwhile, the 1-negative policy led to a total cost of $628,452 (95% CI, $513,592–$752,148), which was significantly lower than that of the 3-negative policy (*P* < .001 for 2-sample T-test). A sensitivity analysis of the contact precaution effectiveness found that the 1-negative policy was consistently lower in cost than the other policies (Supplementary Material online).

**Table 1.** Estimated Value for the Average Annual Cost for 3-Negative, 2-Negative, and 1-Negative Policies for 2017–2019 via Model Simulation^a^

Variable	3-Negative Policy		2-Negative Policy		1-Negative Policy	
	No.	95% CI	No.	95% CI	No.	95% CI
MRSA cases detected	230	193–269	236	200–274	247	207–289
MRSA infection cases detected	26	21–30	26	22–30	27	23–32
MRSA colonized cases detected	204	172–239	210	177–243	220	184–257
Precaution days	1,015	831–1229	986	801–1168	877	713–1055
Precaution days for infections	113	92–136	109	89–130	97	79–117
Precaution days for colonized	902	738–1,093	876	712–1,038	779	634–937
MRSA infection cases treatment cost	$11,710	9,842–13,685	$12,012	10,175–13,940	$12,608	10,540–14,729
MRSA colonized cases treatment cost	$89,042	74,837–104,055	$91,334	77,366–105,993	$95,865	80,140–111,996
MRSA infection cases precaution cost	$237,864	194,614–287,957	$230,962	187,655–273,682	$205,433	167,084–247,092
MRSA colonized cases precaution cost	$364,225	297,999–440,929	$353,657	287,343–419,070	$314,565	255,845–378,354
Total cost	$702,823	577,277–846,605	$687,946*	562,522–812,662	$628,452**	513,592–752,148

Note. MRSA, methicillin-resistant *Staphylococcus aureus.*

a Cost shown in USD, adjusted to 2023.

*P < .005, and **P < .001 compared with 3-negative policy total cost.

## Discussion

We studied the potential cost effectiveness of changing from the current policy of needing 3 consecutive negative MRSA surveillance tests to remove precautions to either a 2-negative clearance policy or a 1-negative clearance policy at the University of Virginia Hospital. To estimate the outcome, we developed a new 2-Mode–Precaution model and calibrated the parameters to data from EHR data. To balance the influence of rising treatment costs due to increased MRSA cases and the reduction in precaution costs resulting from fewer precaution days, we computed the total cost for the 3-negative policy and the other 2 alternative relaxed policies. Our study indicates that a single negative MRSA nares PCR test may provide sufficient evidence to discontinue MRSA contact precautions and that the 1-negative policy may be the most cost-effective option. This research yields 3 key findings: (1) 1-negative and 1-negative policies result in relatively few (<8%) additional MRSA cases when compared to the conventional 3-negative policy; (2) relaxed releasing policies reduce the number of precaution days; and (3) a 1-negative policy leads to significant savings by having substantially lower precaution costs.

This study had several limitations. We assumed that model parameters in the 2-mode–precaution model remained constant throughout the entire period. These parameters may be time varying, with seasonal fluctuations potentially causing significant variations in transmission and MRSA importation rates. We also assumed that the non–precaution-based infection controls were constant, which is true at the University of Virginia Hospital but may not be applicable for other hospitals. Additionally, we only utilized in-hospital MRSA test results to calibrate the 2-Mode–Precaution model due to limited data availability; we did not account for bias in testing patients for MRSA. We also did not factor costs associated with reduced hospital bed capacity. For example, patients on MRSA isolation require placement into single rooms or cohorts, which effectively reduces bed capacity. Additionally, the EHRs employed to construct contact networks only contain interactions where both patients and healthcare providers are present. As a result, interactions exclusively between healthcare providers, such as 2 providers in a breakroom simultaneously, were not recorded and modeled. Moreover, although other individuals like visitors, hospital administrators, and janitorial staff may contribute to MRSA transmission, their involvement is not documented in the EHR. Future research could expand the model to accommodate these time-variant parameters and colonization states. Another limitation of this analysis is that we did not account for healthcare worker fatigue. Repeatedly donning and doffing gowns and gloves may lead to reduced adherence to contact precautions over time (as demonstrated with the COVID-19 pandemic),^23^ thereby potentially reducing the benefits of stricter clearance protocols. Because the cost analysis was hospital-centered, we did not factor potential downstream societal costs associated with increased MRSA-colonized patients in the community as a result of relaxed precaution discontinuation policies. However, they can easily be extended from our proposed model. In this study, we focused on the spread dynamics and economic costs in the University of Virginia Hospital only. The downstream influence of such relaxed contact precaution policy to the community or other care facilities were neither included nor evaluated. Particularly, patients discharged under a 1-negative clearance policy might have led to more transmission in other places, such as nursing homes. A mitigating factor in our study is that most of the additional infections were already under precautions, so this risk was reduced. However, additional work is needed to understand these effects.

## Supporting information

Cui et al. supplementary material Cui et al. supplementary material

## Supplementary material

To view supplementary material for this article, please visit https://doi.org/10.1017/ice.2024.23
