# Supplementary material for: Modeling relaxed policies for discontinuation of methicillin-resistant Staphylococcus aureus contact precautions
Source: Infect Control Hosp Epidemiol. 2024 Feb 26;45(7):833–8. doi: 10.1017/ice.2024.23 (PMC11439595; doi:10.1017/ice.2024.23)
Supplement: Cui et al. supplementary material [file S0899823X24000230sup001.docx]

**Modeling relaxed policies for discontinuation of Methicillin Resistant *Staphylococcus aureus* contact precautions: Supplementary Materials**

Jiaming Cui^1^, Jack Heavey^2^, Leo Lin^2^, Eili Y. Klein MA, PhD^3,4,5^, Gregory R. Madden MD^6^, Costi D. Sifri MD^6,7^, Bryan Lewis PhD^8^, Anil K. Vullikanti PhD^2,8^, B. Aditya Prakash PhD^1*^

1 College of Computing, Georgia Institute of Technology, Atlanta, GA

2 Department of Computer Science, University of Virginia, Charlottesville, VA

3 Center for Disease Dynamics, Economics & Policy, Washington, D.C., USA

4 Department of Emergency Medicine, Johns Hopkins School of Medicine, Baltimore, MD, USA

5 Department of Epidemiology, Johns Hopkins Bloomberg School of Public Health, Baltimore, MD, USA

6 Division of Infectious Diseases & International Health, Department of Medicine, University of Virginia School of Medicine, Charlottesville, VA

7 Office of Hospital Epidemiology/Infection Prevention & Control, UVA Health, Charlottesville, VA

8 Biocomplexity Institute, University of Virginia, Charlottesville, VA

**Number of detected MRSA cases used for calibration:**

The number of detected MRSA cases was obtained from the University of Virginia (UVA) Health System, which contained two different types of tests to diagnose MRSA: culture tests and polymerase chain reaction (PCR) tests. However, since a negative culture test cannot disqualify an individual from contact precautions, we limit our analysis to only positive culture tests and both positive and negative PCR tests. Between January 1, 2015, and December 31, 2019, the UVA Health System conducted 22,825 tests on 15,806 unique patients. Out of these tests, 2,481 yielded positive results, while 20,344 were negative, and 1800 unique patients had at least one positive test.

Tests were administered upon admission to specific hospital wards, such as intensive care units (ICU), based on healthcare provider recommendations, and at regular intervals for patients with extended stays. To accurately calculate the duration of contact precautions, we augment our sequence of test results with hospital admission and discharge data, ensuring that days outside the hospital are not counted. It is important to note that contact precautions do not prevent patients from leaving the hospital. However, if they are under precautions when they are discharged, they are placed under contact precautions again if they are readmitted to the hospital at a later date.

**Construction of heterogeneous contact networks:**

The patient test records are further augmented with colocation data of the entire UVA hospital system. We represent the heterogeneous contact networks with a series of graphs $g_{t}=\left( V,E_{t} \right)$, where $t$ refers to day $t$. In these graphs, nodes can represent patients, healthcare workers (HCWs), or locations. Over 2015 to 2019, the data encompasses 41,216 unique patients, 14,392 unique healthcare workers, and 685 unique locations across all departments within the hospital.

**Supplementary Table 1.** Number of patients, HCWs, and locations across 2015-2019 in UVA dataset

|  | **2015** | **2016** | **2017** | **2018** | **2019** | **Total (unique)** |
| --- | --- | --- | --- | --- | --- | --- |
| Patients | 11,367 | 11,339 | 11,008 | 10,658 | 10,087 | 41,216 |
| Healthcare workers | 3,967 | 3,811 | 8,905 | 8,017 | 7,573 | 14,392 |
| Locations | 574 | 587 | 587 | 533 | 606 | 685 |

However, the electronic health records (EHRs) utilized for constructing the contact networks only contain interactions where both patients and healthcare providers are present. Therefore, interactions between only healthcare providers, such as two providers simultaneously in the breakroom, are not documented. Additionally, while other individuals like visitors, hospital administrators, and janitorial staff can contribute to the spread of MRSA, their interactions are not captured within the EHRs.

**Modeling MRSA spread on heterogeneous contact networks**

There are two pathways in MRSA transmission: (1) Direct contacts via patient-patient contacts, and (2) indirect exposure through contact with contaminated physical surfaces/locations or HCWs who come in contact with infected patients, which can, in turn, spread to healthy people (i.e., exposed). Traditional contact networks can only capture direct contacts between individuals. Therefore, heterogeneous contact networks, which represent different kinds of entities and their interactions, are required to model both pathways in MRSA transmission. Compared to traditional contact networks with only patient-to-patient connections, heterogeneous contact networks can capture pairwise relationships among diverse entities in real-life settings^1^. We construct a series of heterogeneous contact networks for the UVA hospital, comprising three types of entities: patients, healthcare workers (HCWs), and locations to model both MRSA transmission pathways.

One challenge in utilizing heterogeneous contact networks is that traditional infectious disease models cannot be applied directly^2-4^, as locations cannot be classified as susceptible or infectious. Consequently, standard SEIR-type models^5^ are ineffective at capturing complex transmission pathways involving both contaminated surfaces and direct person-to-person contacts and are therefore less suitable for modeling MRSA transmission^6-9^. To address this issue, Jang et al. recently proposed an agent-based model incorporating environmental contamination, referred to as the 2-Mode-SIS model^6^, to capture the spread of healthcare-associated infections (e.g., MRSA) on heterogeneous contact networks. In the 2-Mode-SIS model, the pathogen load (i.e., the amount of MRSA pathogen) for each entity is explicitly represented, and this load can change over time through pathogen exchanges via contacts (edges) between entities. Patients can be in one of two states: susceptible or carriage. The model assumes that each node carries a varying amount of pathogen over time, and pathogen exchanges among patients are driven by both direct (i.e., patient-to-patient) and indirect (HCW-mediated, location mediated, or patient-to-patient-to-patient) exposures. Additionally, the probability of a patient transitioning from a susceptible to a carriage state is determined by a dose-response function, which specifies the probability that the patient becomes infected; this probability increases with the patient's pathogen load^6^. Upon entering the carriage state, patients shed additional pathogens daily, which can then be transferred to neighboring individuals and locations via edges. The shedding continues until the patient recovers (see Supplementary material for more details). Although the 2-Mode-SIS model can work on heterogeneous contact networks and effectively capture MRSA transmission dynamics, it cannot account for contact precautions, as there are no states designated for precautions. Therefore, we extend this 2-Mode-SIS model and propose our 2-Mode-Precaution model.

**2-Mode-Precaution Model:**

We slightly extend the existing 2-Mode-SIS model and propose our 2-Mode-Precaution model in this work. We first introduce the 2-Mode-SIS model in detail in the following section:

With the heterogeneous contact networks $g=\left\{ g_{1},g_{2},\cdots,g_{T} \right\}$ for $T$ days, we can represent the network $g_{t}$ for day $t$ as a contact matrix $A_{t}$, where $A_{t}\left( i,j \right)=1$ means $i$ and $j$ had contact with each other on day $t$, and $A_{t}\left( i,j \right)=0$ otherwise. The node set is consistent across all $g_{t}$ and can represent patients, HCWs, or locations. Each node in the network carries some amount of pathogen load, which changes over time. When two nodes come in contact each other (i.e., $A_{t}\left( i,j \right)=1$), they will exchange some fraction of their pathogen loads with each other. The model use $\tau_{ijt}$ to represent the fraction of pathogen loads transferred from $i$ to $j$ (when $i=j$, $\tau_{ijt}$ will be the fraction of remaining load on node $i$). Using this $\tau_{ijt}$ and $A_{t}$, we can then construct the transfer matrix $R_{t}=\tau_{ijt}A_{t}$. Specifically, in this work, we assume $\tau_{ijt}$ only depends on the node type of $i$ and $j$ (e.g., $\tau_{ijt}$ will be the same for any patient $i$ and patient $j$). Hence have 8 different values of $\tau_{ijt}$: $\tau_{P\to P}$, $\tau_{P\to H}$, $\tau_{P\to L}$, $\tau_{H\to P}$, $\tau_{H\to H}$, $\tau_{H\to L}$, $\tau_{L\to P}$, $\tau_{L\to H}$, where P, H, and L represent patients, HCWs, and locations respectively. With the transfer matrix $R_{t}$, we can use the following equation to capture the change of loads on nodes:

$$l_{t+1}=R_{t}l_{t}+\alpha x_{t}$$

Here, $l_{t}$ is the pathogen load vector on day $t$. $x_{t}$ is the carriage state vector on day $t$ (i.e., $x_{t}\left( i \right)=1$ means $i$ is carriage, and $x_{t}\left( i \right)=0$ otherwise), $\alpha$ is the shedding rate of loads for carriage patients. The first term captures the transfer of loads among nodes via edges. The second describes how the loads increase in carriage patients. Note that we assume only patients can be in the carriage state, so HCWs and locations can carry some pathogen loads but will not be infected or colonized. Note that the column-sums of $R_{t}$ are less than or equal to 1, which implies that the total amount of pathogen cannot increase after transfer (i.e., $\left\| R_{t}l_{t} \right\|_{1}\leq\left\| l_{t} \right\|_{1}$).

There are only 2 states in the 2-Mode-SIS model: suspectable and carriage. In the 2-Mode-SIS model^1^, it uses transmission rate $\beta$ to capture the transfer probability from susceptible to carriage (i.e., $S$ to $C$) and recovery rate $\delta$ to capture the transfer probability from carriage to susceptible (i.e., $C$ to $S$):

$$p_{S\to C}=min\left\{ 1,\beta l_{t}(i) \right\}$$

$$p_{C\to S}=\delta$$

Here, $l_{t}(i)$ is the amount of pathogen loads on node $i$ on day $t$. Intuitively, a higher $l_{t}(i)$ means that node $i$ will be more likely to be infected or colonized, and the 2-Mode-SIS uses a dose-response function to capture it^1^. $\delta$ is the recovery rate.

For patients under contact precautions, we model this process by reducing the fraction of pathogens loads transferred from/to these patients. Specifically, for every patient $i$ under contact precaution, we reduce the $i$th row and column of the pathogen transfer matrix $R_{ijt}$ by an effectiveness parameter $\varphi_{eff}$, which is calibrated on the current 3-negative policy scenario in the UVA hospital.

The model is initialized by assuming each patient may be infected or colonized with probability $p_{initial}$, and these patients will have a pathogen load following the below normal distribution:

$$l_{1}\left( i \right)\sim Normal(\mu_{initial},\sigma_{initial})$$

Based on the 2-Mode-SIS model, we extend it to include six states and build our 2-Mode-Precaution model: four states for patients in the hospital ($S$ - Susceptible, out of contact precaution, $C$ - Carriage, out of contact precaution, $S_{cp}$ - Susceptible, under contact precaution, and $C_{cp}$ - Carriage, under contact precaution) and two states ($S$ and $C$) for patients outside the hospital, or in the community. Note that in the 2-Mode-Precaution model, the carriage and recovery can happen either in hospital (e.g., $S$ to $C$, and $S_{cp}$ to $C_{cp}$) or in community. Thus, we have the following equations:

$$p_{S\to C}=p_{S_{cp}\to C_{cp}}=min\left\{ 1,\beta l_{t}(i) \right\}$$

$$p_{C\to S}=p_{C_{cp}\to S_{cp}}=\delta$$

For a change in contact precaution status, we use 6 calibrated parameters ($p_{S\to S_{cp}}$, $p_{S_{cp}\to S}$, $p_{C\to C_{cp}}$, $p_{C_{cp}\to C}$, $p_{S\to C_{cp}}$, $p_{C_{cp}\to S}$) to capture this change directly.

Here, we provide an example to illustrate how an MRSA patient moves between states: when a patient not under precautions becomes infected or colonized with MRSA, the patient moves from state $S$ to $C$. Next if this patient tests positive for MRSA, the patient moves from $C$ to $C_{cp}$. Subsequently, two scenarios may occur: (1) If the patient recovers from MRSA and moves to state $S_{cp}$, the patient will be released from contact precautions after three negative MRSA tests (i.e., move from $S_{cp}$ to $S$); or (2) If the patient does not recover from MRSA and remains in state $C_{cp}$, the patient may also be released from precautions after three consecutive false-negative MRSA tests (i.e., move from $C_{cp}$ to $C$). As shown in Figure 1, only hospitalized patients can be placed under contact precautions. Patients may be discharged from the hospital while still under contact precaution, and this contact precaution continues if they are readmitted later. However, contact precautions are not enforced when patients are at home/in community.

We also assume that the patients in $S$ in the hospital will be infected or colonized with probability $p_{import}$ at any day $t$ following the widely used MRSA importation modeling setup^2,3^ to capture the MRSA importation. When moving to carriage state, this patient $i$ will move from $S$ to $C$ and have a pathogen load following the below normal distribution:

$$l_{t}\left( i \right)\sim Normal(\mu_{import},\sigma_{import})$$

**Calibration**

Since the current policy to release patients from precautions at the UVA hospital is 3-negative, we can calibrate on the number of detected MRSA cases to obtain the parameters for the 3-negative policy. To infer these parameters, we use the Ensemble Adjustment Kalman Filter (EAKF), which has been widely used for epidemiological models on various healthcare-associated infections, including MRSA, and has demonstrated good performance in identifying model parameters^10-12^. Specifically, we calibrate the number of cases transferred from $C$ to $C_{cp}$ to the number of detected MRSA cases collected from EHR data in the UVA hospital. Note that although patients can also become MRSA carriers outside of the hospital, we do not have such data, so we only use the number of detected MRSA cases in the UVA hospital for calibration. We list the value of calibrated parameters in Supplementary Table 2.

**Supplementary Table 2.** Value of calibrated parameters

| year | 2015 | 2016 | 2017 | 2018 | 2019 | Range |
| --- | --- | --- | --- | --- | --- | --- |
| $p_{initial}$ | 0.0050(SDs: 8.9744$\times{10}^{-5}$) | 0.0055(SDs: 8.8917$\times{10}^{-5}$) | 0.0050 (SDs: 8.7139$\times{10}^{-5}$) | 0.0046 (SDs: 9.6112$\times{10}^{-5}$) | 0.0050 (SDs: 8.9608$\times{10}^{-5}$) | [0,0.01]^13^ |
| $\mu_{initial}$ | 4.8824(SDs: 0.0912) | 5.2399(SDs: 0.0926) | 4.5661(SDs: 0.0854) | 4.8548 (SDs: 0.0866) | 6.0005 (SDs: 0.0933) | [0,10] |
| $\sigma_{initial}$ | 5.3234(SDs: 0.0940) | 5.1911(SDs: 0.0896) | 5.1555(SDs: 0.0919) | 5.1254 (SDs: 0.0816) | 5.5318 (SDs: 0.0895) | [0,10] |
| $p_{import}$ | 0.0014(SDs: 4.3755$\times{10}^{-5}$) | 0.0015(SDs: 4.4478$\times{10}^{-5}$) | 0.0018(SDs: 4.2256$\times{10}^{-5}$) | 0.0020 (SDs: 4.4401$\times{10}^{-5}$) | 0.0041 (SDs: 4.2741$\times{10}^{-5}$) | [0,0.005]^13^ |
| $\mu_{import}$ | 4.8770(SDs: 0.0874) | 4.6360(SDs: 0.0902) | 5.0916(SDs: 0.0904) | 5.0647 (SDs: 0.0921) | 5.002 (SDs: 0.0848) | [0,10] |
| $\sigma_{import}$ | 4.7561(SDs: 0.0908) | 4.9666(SDs: 0.0943) | 4.8430(SDs: 0.0879) | 5.1502 (SDs: 0.0884) | 4.8371 (SDs: 0.0939) | [0,10] |
| $\varphi_{eff}$ | 0.2460(SDs: 0.0008) | 0.2488(SDs: 0.0009) | 0.2517(SDs: 0.0009) | 0.2505(SDs: 0.0010) | 0.2491(SDs: 0.0009) | [0.9,1]^13^ |
| $\alpha$ | 0.5933(SDs: 0.0182) | 0.6721(SDs: 0.0189) | 0.5933(SDs: 0.0186) | 0.3314 (SDs: 0.0175) | 1.4072 (SDs: 0.0178) | [0,2]^13^ |
| $\beta$ | 0.0124(SDs: 0.0004) | 0.0103(SDs: 0.0004) | 0.0095(SDs: 0.0004) | 0.0140 (SDs: 0.0005) | 0.0466 (SDs: 0.0006) | [0,0.05]^6^ |
| $\delta$ | 0.0644(SDs: 0.0009) | 0.0583(SDs: 0.0009) | 0.0671(SDs: 0.0009) | 0.0609 (SDs: 0.0010) | 0.0304 (SDs: 0.0009) | [0,0.01]^6^ |
| $\tau_{P\to P}$ | 0.0103(SDs: 0.0001) | 0.0100(SDs: 0.0001) | 0.0090(SDs: 0.0001) | 0.0099 (SDs: 0.0001) | 0.0107 (SDs: 0.0001) | [0,0.02]^6^ |
| $\tau_{P\to H}$ | 0.0105(SDs: 0.0001) | 0.0104(SDs: 0.0001) | 0.0099(SDs: 0.0001) | 0.0099 (SDs: 0.0001) | 0.0092 (SDs: 0.0001) | [0,0.02]^6^ |
| $\tau_{P\to L}$ | 0.0101(SDs: 0.0001) | 0.0092(SDs: 0.0001) | 0.0103(SDs: 0.0001) | 0.0103 (SDs: 0.0001) | 0.0110 (SDs: 0.0001) | [0,0.02]^6^ |
| $\tau_{H\to P}$ | 0.0108(SDs: 0.0001) | 0.0103(SDs: 0.0001) | 0.0099(SDs: 0.0001) | 0.0088 (SDs: 0.0001) | 0.0089 (SDs: 0.0001) | [0,0.02]^6^ |
| $\tau_{H\to H}$ | 0.0010(SDs: 1.7598$\times{10}^{-5}$) | 0.0009(SDs: 1.8232$\times{10}^{-5}$) | 0.0010(SDs: 1.7919$\times{10}^{-5}$) | 0.0010 (SDs: 1.8208$\times{10}^{-5}$) | 0.0010 (SDs: 1.7670$\times{10}^{-5}$) | [0,0.002]^6^ |
| $\tau_{H\to L}$ | 0.0103(SDs: 0.0001) | 0.0099(SDs: 0.0001) | 0.0101(SDs: 0.0001) | 0.0106 (SDs: 0.0001) | 0.0096 (SDs: 0.0001) | [0,0.02]^6^ |
| $\tau_{L\to P}$ | 0.0104(SDs: 0.0001) | 0.0096(SDs: 0.0001) | 0.0.99(SDs: 0.0001) | 0.0096 (SDs: 0.0001) | 0.0091 (SDs: 0.0001) | [0,0.02]^6^ |
| $\tau_{L\to H}$ | 0.0048(SDs: 8.9161$\times{10}^{-5}$) | 0.0051(SDs: 8.8080$\times{10}^{-5}$) | 0.0048(SDs: 8.5947$\times{10}^{-5}$) | 0.0047 (SDs: 8.7892$\times{10}^{-5}$) | 0.0047 (SDs: 8.6480$\times{10}^{-5}$) | [0,0.01]^6^ |
| $p_{S\to S_{cp}}$ | 0.0186(SDs: 0.0004) | 0.0192(SDs: 0.0003) | 0.0185(SDs: 0.0004) | 0.0204 (SDs: 0.0003) | 0.0189 (SDs: 0.0003) | [0,0.04] |
| $p_{S_{cp}\to S}$ | 0.2003(SDs: 0.0018) | 0.1906(SDs: 0.0018) | 0.1920(SDs: 0.0019) | 0.1986 (SDs: 0.0018) | 0.1951 (SDs: 0.0018) | [0.1,0.3] |
| $p_{C\to C_{cp}}$ | 0.4432(SDs: 0.0053) | 0.4445(SDs: 0.0053) | 0.4709(SDs: 0.0056) | 0.4663 (SDs: 0.0048) | 0.6322 (SDs: 0.0052) | [0.2,0.8] |
| $p_{C_{cp}\to C}$ | 0.1011(SDs: 0.0009) | 0.0969(SDs: 0.0009) | 0.1002(SDs: 0.0009) | 0.0991 (SDs: 0.0009) | 0.1111 (SDs: 0.0009) | [0.05,0.15] |
| $p_{S\to C_{cp}}$ | 0.0009(SDs: 1.7344$\times{10}^{-5}$) | 0.0009(SDs: 1.9443$\times{10}^{-5}$) | 0.0009(SDs: 1.7605$\times{10}^{-5}$) | 0.0010 (SDs: 1.6815$\times{10}^{-5}$) | 0.0010 (SDs: 1.7355$\times{10}^{-5}$) | [0,0.002] |
| $p_{C_{cp}\to S}$ | 0.0010(SDs: 1.7680$\times{10}^{-5}$) | 0.0009(SDs: 1.6958$\times{10}^{-5}$) | 0.0010(SDs: 1.7698$\times{10}^{-5}$) | 0.0010 (SDs: 1.7577$\times{10}^{-5}$) | 0.0009 (SDs: 1.9056$\times{10}^{-5}$) | [0,0.002] |

**Parameter adjustment method:**

When altering the releasing policy from 3-negative to 2-negative, the most clinically significant parameter changes in the 2-Mode-Precaution model should be
$p_{C_{cp}\to C}$ (the transition probability from $C_{cp}$ to $C$) and
$p_{S_{cp}\to S}$ (transition probability from $S_{cp}$ to $S$). Other parameters should not change significantly, assuming that other non-precaution-based infection controls and prevention measures remain constant (e.g., terminal room disinfection, standard hand hygiene practices, antimicrobial use). We use $p_{C_{cp}\to C}^{3neg}$ to represent the parameter for the 3-negative policy, and $p_{C_{cp}\to C}^{2neg}$ to represent the parameter for the 2-negative policy (similarly, $p_{S_{cp}\to S}^{3neg}$ and $p_{S_{cp}\to S}^{2neg}$). Next, we will demonstrate how to utilize the MRSA PCR test records from EHR data to adjust from from $p_{C_{cp}\to C}^{3neg}$ to estimate $p_{C_{cp}\to C}^{2neg}$.

First, let's focus on $p_{C_{cp}\to C}$. Under the 3-negative policy, only patients with three consecutive negative tests will be released. However, under the 2-negative policy, patients with the first two tests negative will also be released without a third test. We use $n_{0}$ to represent the number of records with three consecutive negative tests in EHR data (i.e., negative-negative-negative), and $n_{1}$ to represent the number of records with two negative test results followed by one positive result (i.e., negative-negative-positive). With the 2-negative policy, $n_{1}$ more patients will be released. Hence, $p_{C_{cp}\to C}$ for the 2-negative policy will be $p_{C_{cp}\to C}^{2neg}\approx min\{1,\frac{n_{0}+n_{1}}{n_{0}}p_{C_{cp}\to C}^{3neg}\}$. Intuitively, the relaxed policy will result in a higher $p_{C_{cp}\to C}^{2neg}$ than $p_{C_{cp}\to C}^{3neg}$, since MRSA infectious cases are released with a higher probability due to one fewer test. To extract results of MRSA tests, we parse each patient's EHR and extract MRSA PCR nares and culture test results, constructing our dataset using the date the test was taken and the result of the test. As described above, we exclude negative culture tests, as a negative culture test does not preclude the presence of MRSA and is thus not sufficient to clear contact precautions at the  UVA hospital.

Next, let's focus on $p_{S_{cp}\to S}$. Under the 3-negative policy, patients must wait for three consecutive negative tests to be released. However, under the 2-negative policy, patients only need to wait for two consecutive negative tests, which indicates a shorter waiting time and hence a larger transfer probability. We use $d_{3}$ to capture the average number of days to obtain three consecutive negative tests, and $d_{2}$ to capture the average days to obtain the first two consecutive negative results after the initial positive test. By assuming that the average days follow a geometric distribution parameterized by $p_{S_{cp}\to S}$, then $p_{S_{cp}\to S}^{3neg}$ should be proportional to $\frac{1}{d_{3}}$, and $p_{S_{cp}\to S}^{2neg}$ should be proportional to $\frac{1}{d_{2}}$. Therefore, we have $p_{S_{cp}\to S}^{2neg}\approx min\{1,\frac{d_{3}}{d_{2}}p_{S_{cp}\to S}^{3neg}\}$. Intuitively, the relaxed 2-negative policy will result in a higher $p_{S_{cp}\to S}^{2neg}$than $p_{S_{cp}\to S}^{3neg}$, since patients are released out of precaution faster with a higher probability.

For the calculation of $d_{3}$, we analyze the number of instances in patient EHRs where an individual tests positive for MRSA and then receives three consecutive negative MRSA tests as their next three tests on record. We then count the number of days between the first positive test and the third negative test, excluding days when the patient was not present in the hospital (as calculated by their admit and discharge days). This value is then averaged across all instances of a positive test followed by three negative tests. Similarly, for $d_{2}$, we use the same subsets of patients that have a positive test followed by three negative tests in order to perform a consistent comparison. However, we then take the number of days between the positive test and the second negative test, again discounting time when a patient has left the hospital, and average this over the entire data set. Due to this methodology, we will always have $d_{2}<d_{3}$, as hospital policy requires at least 24 hours between tests.

In summary, we can use the parameters calibrated for the current 3-negative policy in the UVA hospital to estimate parameters for the 2-negative policy with the following approach: First, we calibrate the 2-Mode-Precaution model to the number of detected cases in the UVA hospital, obtaining the parameters for the 3-negative policy. Next, we apply the parameter adjustment method to estimate the parameters for the 2-negative policy. We then run simulations with the adjusted parameters to estimate the 2-negative policy scenario. Similarly, we can also adjust the calibrated 3-negative parameters to estimate the 1-negative policy scenario. We also list the values of $n_{0}, n_{1}, n_{2}$ and $d_{3},d_{2},d_{1}$ in Supplementary Table 3.

**Supplementary Table 3.** Value of $n_{0}, n_{1}, n_{2}$ and $d_{3},d_{2},d_{1}$

| year | **2015** | **2016** | **2017** | **2018** | **2019** |
| --- | --- | --- | --- | --- | --- |
| $n_{0}$ | 81 | 138 | 121 | 122 | 278 |
| $n_{1}$ | 12 | 9 | 9 | 14 | 41 |
| $n_{2}$ | 30 | 21 | 15 | 28 | 78 |
| $d_{3}$ | 155.5 | 134.25 | 101.75 | 15.66 | 169.5 |
| $d_{2}$ | 154.5 | 97.75 | 86 | 15 | 140 |
| $d_{1}$ | 153.5 | 74.75 | 21.75 | 4.33 | 115.625 |

**Effectiveness of calibration procedure:**

Here, we demonstrate the effectiveness of the calibration procedure from two perspectives: First, we conduct a synthetic experiment to demonstrate our ability to "recover" synthetic parameters by calibrating on the simulated curves generated using these parameters. Second, we illustrate that our simulated curves, derived from the calibrated parameters, align closely with the real-world number of detected MRSA cases.

Here, we set up 5 different "scenarios" for synthetic experiments. In each scenario, we set “ground truth” parameters and run simulations with these ground-truth parameters to generate a synthetic detected MRSA cases curves. Next, we use each synthetic MRSA case curve for calibration and obtain the calibrated parameters. If the calibrated parameters closely align with the ground truth parameters we initially set (i.e., we can "recover" these parameters through calibration), we conclude that the calibration process is effective.

**Supplementary Figure 1.** The calibration procedure effectively "recovers" the synthetic parameters by calibrating on the simulated curve generated using these synthetic parameters. Left: red dots represent the ground-truth values of synthetic parameters, while blue dots and error bars represent the mean value and 95% confidence interval of calibrated parameters. We can see our calibrated parameters closely align with their ground-truth parameters. Right: the error between our estimated parameters and ground-truth parameters.

We show our results in Supplementary Figure 1. As shown in the figure, we compare the ground truth parameter values (red dots) with the calibrated parameter values (blue dots and error bars, representing mean value and 95% confidence interval) on the left. Our calibrated parameters closely align with the ground truth parameters (in fact, the ground truth parameters always fall within the 95% confidence interval of the calibrated parameters). We also display the error between calibrated parameters and ground-truth parameters on the right. By selecting multiple values for the ground truth parameters, we show calibration is effective over a broad range of ground truth parameters. It is worth noting that the absolute value of errors remains small. Therefore, we can conclude that our calibration procedure effectively recovers the synthetic parameters across a wide range of parameters.

Next, we show that the calibration procedure works well on the number of MRSA cases from EHR data. Here, we use the cumulative values of the number of MRSA cases for calibration to obtain the calibrated parameters. We then simulate with these calibrated parameters to get the simulated curves. If the simulated curves are close to the observed values, we can conclude that the calibration procedure works well on the actual number of MRSA cases.

We show the results in Supplementary Figure 2. Here, black dots are the cumulative number of detected MRSA cases under the 3-negative policy in UVA hospital now. Blue curves and shaded regions are our simulated curves' mean value and 95% confidence interval. Note that the blue curve closely aligns to  the blue dots, and most black dots are within the confidence interval of the simulated curves (blue shadow area). Hence, the calibrated parameters fit the real-world number of detected MRSA cases well.

**Supplementary Figure 2.** Our calibrated parameters fit the real-world number of detected MRSA cases well. Black dots represent the cumulative number of detected MRSA cases. The blue curves and shaded regions represent the mean value and 95% confidence interval for the cumulative number of detected MRSA cases based on the calibrated parameters. The x-axis is the date, while the y-axis is the cumulative value for the number of detected MRSA cases. Panels (a), (b), (c) correspond to 2017, 2018, 2019, respectively.

**Additional results:**

As shown in Supplementary Figure 3, the 2-negative policy leads to 3.62% more MRSA cases (95% CI: -13.3% to 18.9%, $p<0.001$) than the 3-negative policy, while the 1-negative policy results in 7.06 % more MRSA cases (95% CI: -10.7% to 25.4%, $p<0.001$) over the 2015-2016 period.

**Supplementary Figure 3.** The 2-negative and 1-negative policies lead to more new detected MRSA cases compared to the current 3-negative policy. (a) Blue solid dots and error bars indicate the mean value and 95% confidence interval for detected MRSA cases per 10,000 patient-day under the 3-negative policy, as determined by calibration. The red dashed and green dashdot dots and error bars represent the estimated mean values and 95% confidence intervals for the number of detected MRSA cases per 10,000 patient-days under the 2-negative and 1-negative policies, respectively. To demonstrate the differences between the 3-negative policy and the 2-negative and 1-negative policies, we employ the 2-sample t-test (*$p<0.05$, **$p<0.01$, ***$p<0.001$). The x-axis is the number of detected MRSA cases per 10,000 patient-day, and the y-axis corresponds to 2015 and 2016. (b-c) The blue solid curves and shaded regions represent the mean value and 95% confidence interval for the cumulative number of detected MRSA cases under the 3-negative policy as determined by calibration. The red dashed and green dashdot curves and shaded areas represent the estimated number of detected MRSA cases for 2-negative and 1-negative policies, respectively. The x-axis is the date, while the y-axis is the cumulative value for detected MRSA cases. Panels (b), (c) correspond to 2015, 2016, respectively.

**Supplementary Figure 4.** Most MRSA cases are under contact precautions. The shaded areas represent the weekly number of in-hospital patients in C and Ccp states under the 1-negative policy. The gray and purple sections correspond to cases that are under or not under contact precautions, respectively. Panels (a), (b) correspond to 2015, 2016, respectively.

As shown in Supplementary Figure 4, 73.1% (95% CI: 71.23%-74.77%) of carriage patients from 2015 to 2016 remain under contact precautions.

**Supplementary Figure 5.** Distribution for contact precaution durations related to MRSA. The blue solid, red dashed, and green dashdot curves represent the distribution for 3-, 2-, and 1-negative policies, respectively. The x-axis is the contact precaution durations in days, while the y-axis is the probability. Panels (a), (b) correspond to 2015, 2016, respectively.

As shown in Supplementary Figure 5, the average duration time under contact precautions for 2015-2016 for 3-negative, 2-negative, and 1-negative policies are 6.51 (SDs: 5.08), 6.15 (SDs: 4.63), 5.71 (SDs: 4.24) days.

**Supplementary Table 4.** Estimated value for the average annual cost for 3-negative, 2-negative, and 1-negative policy for 2015-2019 via simulation (in USD)

|  | **3-negative** | **2-negative** | **1-negative** |
| --- | --- | --- | --- |
| Number of detected MRSA cases | 231 (95% CI: 195-269) | 237 (95% CI: 200-275) | 248 (95% CI: 207-290) |
| Number of detected MRSA infection cases | 26 (95% CI: 22-30) | 26 (95% CI: 22-30) | 28 (95% CI: 23-32) |
| Number of detected MRSA colonized cases | 205 (95% CI: 173-239) | 211 (95% CI: 178-244) | 220 (95% CI: 184-258) |
| Number of precaution days | 1,063 (95% CI: 866-1,286) | 1,035 (95% CI: 844-1,241) | 953 (95% CI: 773-1,139) |
| Number of precaution days (for infections) | 118 (95% CI: 96-143) | 115 (95% CI: 94-138) | 106 (95% CI: 86-126) |
| Number of precaution days (for colonized) | 945 (95% CI: 770-1,143) | 920 (95% CI: 750-1,103) | 847 (95% CI: 687-1,013) |
| MRSA infection cases treatment cost | 11,760 (95% CI: 9,931-13,710) | 12,098 (95% CI: 10,207-13,991) | 12,631 (95% CI: 10,553-14,770) |
| MRSA colonized cases treatment cost | 89,420 (95% CI: 75,511-104,247) | 91,990 (95% CI: 77,612-106,380) | 96,043 (95% CI: 80,243-112,307) |
| MRSA infection cases precaution cost | 249,160 (95% CI: 202,896-301,337) | 242,452 (95% CI: 197,803-290,697) | 223,330 (95% CI: 181,040-266,995) |
| MRSA colonized cases precaution cost | 381,521 (95% CI: 310,681-461,416) | 371,250 (95% CI: 302,882-445,124) | 341,970 (95% CI: 277,213-408,831) |
| Total cost | 731,843 (95% CI: 599,003-880,688) | 717,771 (95% CI: 588,489-856,169)** | 673,955 (95% CI: 549,032-802,880)*** |

** indicates that p<0.005, and *** indicates that p<0.001 compared with 3-negative policy total cost

As shown in Supplementary Table 3 for 2015-2019, the 2-negative policy total cost ($447,518 (95% CI: $371,925-$526,145)) is marginally higher than the 3-negative policy ($446,283 (95% CI: $370,590-$529,359)). However, this difference is not statistically significant ($p=0.3$ for 2-sample T-test). In contrast, the 1-negative policy exhibits a total cost ($442,324 (95% CI: $364,918-$522,114)) that is significantly lower than the 3-negative policy ($p<0.005$ for 2-sample T-test).

**Sensitive analysis of contact precaution effectiveness:**

As described in the main article and previous text, we use a parameter $\varphi_{eff}$ to capture the contact precaution effectiveness, which assumes that when a patient i is put under contact precaution, the pathogen load transferred from node $i$ to another node $j$, or from node $j$ to $i$ via contacts also reduce by $\varphi_{eff}$. In the experiments shown in the main article, we calibrated the 2-Mode-Precaution model to the current 3-negative policy situation in UVA hospital to get the value of $\varphi_{eff}$. In addition to the experiments shown in the main article, we also perform a sensitivity analysis by assuming different $\varphi_{eff}$ values to see how our experimental results can be influenced by different contact precaution effectiveness.

Specifically, we run the new experiments with other parameters fixed (same as calibration results) but change $\varphi_{eff}$ to 0.25, 0.5, 0.75, respectively. Results are shown in Supplementary Table 5-7. As shown in the tables, with lower $\varphi_{eff}$ (which corresponds to less pathogen load transferred via contacts and higher contact precaution effectiveness), although both the number of detected MRSA cases and precaution days are decreasing for all policies, the 1-negative policy still consistently outperforms the current 3-negative policy in total cost.

**Supplementary Table 5.** Estimated value for the average annual cost for 3-negative, 2-negative, and 1-negative policy for 2017-2019 with $\varphi_{eff}=0.25$ via simulation (in USD)

|  | **3-negative** | **2-negative** | **1-negative** |
| --- | --- | --- | --- |
| Number of detected MRSA cases | 230 (95% CI: 194-266) | 235 (95% CI: 197-273) | 247 (95% CI: 210-288) |
| Number of detected MRSA infection cases | 26 (95% CI: 22-30) | 26 (95% CI: 22-30) | 27 (95% CI: 23-32) |
| Number of detected MRSA colonized cases | 204 (95% CI: 173-237) | 209 (95% CI: 175-242) | 220 (95% CI: 187-256) |
| Number of precaution days | 1,008 (95% CI: 823-1,218) | 981 (95% CI: 796-1,176) | 877 (95% CI: 711-1,056) |
| Number of precaution days (for infections) | 112 (95% CI: 91-135) | 109 (95% CI: 88-131) | 97 (95% CI: 79-117) |
| Number of precaution days (for colonized) | 896 (95% CI: 732-1,083) | 872 (95% CI: 708-1,046) | 779 (95% CI: 632-939) |
| MRSA infection cases treatment cost | 11,710 (95% CI: 9,902-13,567) | 11,983 (95% CI: 10,022-13,889) | 12,608 (95% CI: 10,726-14,678) |
| MRSA colonized cases treatment cost | 89,041 (95% CI: 75,292-103,157) | 91,115 (95% CI: 76,203-105,605) | 95,866 (95% CI: 81,554-111,608) |
| MRSA infection cases precaution cost | 236,085 (95% CI: 192,845-285,413) | 229,873 (95% CI: 186,550-275,650) | 205,434 (95% CI: 166,694-247,494) |
| MRSA colonized cases precaution cost | 361,501 (95% CI: 295,290-437,033) | 351,988 (95% CI: 285,651-422,084) | 314,567 (95% CI: 255,247-378,970) |
| Total cost | 698,318 (95% CI: 573,314-839,149) | 684,940 (95% CI: 558,410-817,206)** | 628,456 (95% CI: 514,204-752,727)*** |

** indicates that p<0.005, and *** indicates that p<0.001 compared with 3-negative

**Supplementary Table 6.** Estimated value for the average annual cost for 3-negative, 2-negative, and 1-negative policy for 2017-2019 with $\varphi_{eff}=0.5$ via simulation (in USD)

|  | **3-negative** | **2-negative** | **1-negative** |
| --- | --- | --- | --- |
| Number of detected MRSA cases | 233 (95% CI: 199-270) | 240 (95% CI: 204-280) | 253 (95% CI: 212-294) |
| Number of detected MRSA infection cases | 26 (95% CI: 22-30) | 27 (95% CI: 23-31) | 28 (95% CI: 24-33) |
| Number of detected MRSA colonized cases | 207 (95% CI: 177-240) | 213 (95% CI: 182-249) | 225 (95% CI: 189-261) |
| Number of precaution days | 1,027 (95% CI: 812-1,233) | 1,005 (95% CI: 808-1,196) | 900 (95% CI: 731-1,085) |
| Number of precaution days (for infections) | 114 (95% CI: 90-137) | 112 (95% CI: 90-133) | 100 (95% CI: 81-120) |
| Number of precaution days (for colonized) | 913 (95% CI: 722-1,096) | 894 (95% CI: 719-1,063) | 800 (95% CI: 650-965) |
| MRSA infection cases treatment cost | 11,888 (95% CI: 10,123-13,753) | 12,219 (95% CI: 10,412-14,274) | 12,879 (95% CI: 10,811-14,968) |
| MRSA colonized cases treatment cost | 90,388 (95% CI: 76,972-104,572) | 92,908 (95% CI: 79,168-108,534) | 97,928 (95% CI: 82,200-113,811) |
| MRSA infection cases precaution cost | 240,603 (95% CI: 190,328-288,906) | 235,512 (95% CI: 189,418-280,213) | 210,921 (95% CI: 171,347-254,250) |
| MRSA colonized cases precaution cost | 368,419 (95% CI: 291,436-442,382) | 360,624 (95% CI: 290,043-429,071) | 322,968 (95% CI: 262,372-389,315) |
| Total cost | 711,278 (95% CI: 568,843-849,591) | 701,244 (95% CI: 569,024-832,070)** | 644,675 (95% CI: 526,712-772,320)*** |

** indicates that p<0.005, and *** indicates that p<0.001 compared with 3-negative

**Supplementary Table 7.** Estimated value for the average annual cost for 3-negative, 2-negative, and 1-negative policy for 2017-2019 with $\varphi_{eff}=0.75$ via simulation (in USD)

|  | **3-negative** | **2-negative** | **1-negative** |
| --- | --- | --- | --- |
| Number of detected MRSA cases | 238 (95% CI: 199-280) | 245 (95% CI: 208-287) | 258 (95% CI: 217-298) |
| Number of detected MRSA infection cases | 26 (95% CI: 22-31) | 27 (95% CI: 23-32) | 29 (95% CI: 24-33) |
| Number of detected MRSA colonized cases | 212 (95% CI: 177-249) | 217 (95% CI: 185-255) | 229 (95% CI: 193-265) |
| Number of precaution days | 1,056 (95% CI: 849-1,281) | 1,034 (95% CI: 857-1,246) | 925 (95% CI: 751-1,119) |
| Number of precaution days (for infections) | 117 (95% CI: 94-142) | 115 (95% CI: 95-138) | 103 (95% CI: 83-124) |
| Number of precaution days (for colonized) | 939 (95% CI: 755-1,138) | 919 (95% CI: 762-1,107) | 823 (95% CI: 667-995) |
| MRSA infection cases treatment cost | 12,144 (95% CI: 10,157-14,272) | 12,465 (95% CI: 10,599-14,636) | 13,155 (95% CI: 11,083-15,207) |
| MRSA colonized cases treatment cost | 92,335 (95% CI: 77,230-108,521) | 94,780 (95% CI: 80,588-111,289) | 100,028 (95% CI: 84,273-115,625) |
| MRSA infection cases precaution cost | 247,460 (95% CI: 199,033-300,067) | 242,311 (95% CI: 200,735-291,862) | 216,818 (95% CI: 175,877-262,203) |
| MRSA colonized cases precaution cost | 378,919 (95% CI: 304,765-459,472) | 371,035 (95% CI: 307,372-446,909) | 331,998 (95% CI: 269,308-401,493) |
| Total cost | 730,839 (95% CI: 591,169-882,310) | 720,571 (95% CI: 599,278-864,673)** | 661,979 (95% CI: 540,524-794,503)*** |

** indicates that p<0.005, and *** indicates that p<0.001 compared with 3-negative

We also perform another analysis by assuming that the disinfection (e.g., cleaning practices) are also used during the contact precaution process, which will reduce the pathogen load on patients under contact precaution by $\varphi_{disinfection}$ every day. Following the previous studies, we have $\varphi_{disinfection}=0.84$.^14^ Therefore, we run the new experiments with other parameters fixed (same as calibration results, including the $\varphi_{edge}$) and add the clearing practice into modeling by assuming $\varphi_{disinfection}=0.84$. Results are shown in Supplementary Table 8. Specifically, with the cleaning practice, although both the number of detected MRSA cases and precaution days are decreasing for all policies, the 1-negative policy still consistently outperforms the current 3-negative policy in total cost.

**Supplementary Table 8.** Estimated value for the average annual cost for 3-negative, 2-negative, and 1-negative policy for 2017-2019 with cleaning practice via simulation (in USD)

|  | **3-negative** | **2-negative** | **1-negative** |
| --- | --- | --- | --- |
| Number of detected MRSA cases | 229 (95% CI: 176-266) | 236 (95% CI: 200-277) | 247 (95% CI: 209-287) |
| Number of precaution days | 1,002 (95% CI: 824-1,208) | 985 (95% CI: 801-1,193) | 872 (95% CI: 710-1,059) |
| MRSA detected cases cost | 236,662 (95% CI: 202,495-274,756) | 243,477 (95% CI: 206,802-286,103) | 255,088 (95% CI: 215,235-296,290) |
| Precaution cost | 196,256 (95% CI: 161,266-236,527) | 192,810 (95% CI: 156,834-233,661) | 170,702 (95% CI: 138,989-207,457) |
| Total cost | 432,918 (95% CI: 363,761-511,283) | 436,286 (95% CI: 363,636-519,764) | 425,789 (95% CI: 354,223-503,747)*** |

|  | **3-negative** | **2-negative** | **1-negative** |
| --- | --- | --- | --- |
| Number of detected MRSA cases | 229 (95% CI: 196-266) | 236 (95% CI: 200-277) | 247 (95% CI: 209-287) |
| Number of detected MRSA infection cases | 25 (95% CI: 22-30) | 26 (95% CI: 22-31) | 27 (95% CI: 23-32) |
| Number of detected MRSA colonized cases | 204 (95% CI: 175-237) | 210 (95% CI: 178-247) | 220 (95% CI: 185-255) |
| Number of precaution days | 1,002 (95% CI: 824-1,208) | 985 (95% CI: 801-1,193) | 872 (95% CI: 710-1,059) |
| Number of precaution days (for infections) | 111 (95% CI: 91-134) | 109 (95% CI: 89-132) | 97 (95% CI: 79-118) |
| Number of precaution days (for colonized) | 891 (95% CI: 732-1,074) | 875 (95% CI: 712-1,061) | 775 (95% CI: 631-942) |
| MRSA infection cases treatment cost | 11,692 (95% CI: 10,004-13,574) | 12,029 (95% CI: 10,217-14,135) | 12,602 (95% CI: 10,634-14,638) |
| MRSA colonized cases treatment cost | 88,902 (95% CI: 76,067-103,212) | 91,462 (95% CI: 77,685-107,475) | 95,824 (95% CI: 80,853-111,301) |
| MRSA infection cases precaution cost | 234,820 (95% CI: 192,954-283,003) | 230,696 (95% CI: 187,651-279,575) | 204,244 (95% CI: 166,299-248,222) |
| MRSA colonized cases precaution cost | 359,564 (95% CI: 295,457-433,344) | 353,249 (95% CI: 287,337-428,093) | 312,744 (95% CI: 254,643-380,085) |
| Total cost | 694,960 (95% CI: 574,468-833,112) | 687,417 (95% CI: 562,874-829,255)** | 625,395 (95% CI: 512,412-754,224)*** |

** indicates that p<0.005, and *** indicates that p<0.001 compared with 3-negative

**References:**

1. Jang H, Justice S, Polgreen PM, Segre AM, Sewell DK, Pemmaraju SV. Evaluating architectural changes to alter pathogen dynamics in a dialysis unit: for the CDC MInD-healthcare group. 2019:961-968.

2. Pei S, Morone F, Liljeros F, Makse H, Shaman JL. Inference and control of the nosocomial transmission of methicillin-resistant Staphylococcus aureus. *Elife*. 2018;7:e40977.

3. Pei S, Liljeros F, Shaman J. Identifying asymptomatic spreaders of antimicrobial-resistant pathogens in hospital settings. *Proceedings of the National Academy of Sciences*. 2021;118(37):e2111190118.

4. Pei S, Kandula S, Shaman J. Differential effects of intervention timing on COVID-19 spread in the United States. *Science advances*. 2020;6(49):eabd6370.

5. Li R, Pei S, Chen B, et al. Substantial undocumented infection facilitates the rapid dissemination of novel coronavirus (SARS-CoV-2). *Science*. 2020;368(6490):489-493.

1. Roth VR, Longpre T, Coyle D, et al. Cost analysis of universal screening vs. risk factor-based screening for methicillin-resistant Staphylococcus aureus (MRSA). *PLOS ONE*. 2016;11(7):e0159667.

2. Wagner R, Agusto FB. Transmission dynamics for Methicilin-resistant Staphalococous areus with injection drug user. *BMC Infectious Diseases*. 2018;18(1):1-18.

3. Wang X, Panchanathan S, Chowell G. A data-driven mathematical model of CA-MRSA transmission among age groups: evaluating the effect of control interventions. *PLoS Computational Biology*. 2013;9(11):e1003328.

4. Macal CM, North MJ, Collier N, et al. Modeling the transmission of community-associated methicillin-resistant Staphylococcus aureus: a dynamic agent-based simulation. *Journal of Translational Medicine*. 2014;12(1):1-12.

5. Hethcote HW. The mathematics of infectious diseases. *SIAM review*. 2000;42(4):599-653.

6. Jang H, Justice S, Polgreen PM, Segre AM, Sewell DK, Pemmaraju SV. Evaluating architectural changes to alter pathogen dynamics in a dialysis unit: for the CDC MInD-healthcare group. 2019:961-968.

7. Plipat N, Spicknall IH, Koopman JS, Eisenberg JN. The dynamics of methicillin-resistant Staphylococcus aureusexposure in a hospital model and the potential for environmental intervention. *BMC infectious diseases*. 2013;13(1):1-11.

8. Li S, Eisenberg JN, Spicknall IH, Koopman JS. Dynamics and control of infections transmitted from person to person through the environment. *American Journal of Epidemiology*. 2009;170(2):257-265.

9. Giardina J, Bilinski A, Fitzpatrick MC, et al. Model-estimated association between simulated US elementary school–related SARS-CoV-2 transmission, mitigation interventions, and vaccine coverage across local incidence levels. *JAMA Network Open*. 2022;5(2):e2147827-e2147827.

10. Li R, Pei S, Chen B, et al. Substantial undocumented infection facilitates the rapid dissemination of novel coronavirus (SARS-CoV-2). *Science*. 2020;368(6490):489-493.

11. Pei S, Liljeros F, Shaman J. Identifying asymptomatic spreaders of antimicrobial-resistant pathogens in hospital settings. *Proceedings of the National Academy of Sciences*. 2021;118(37):e2111190118.

12. Pei S, Kandula S, Shaman J. Differential effects of intervention timing on COVID-19 spread in the United States. *Science Advances*. 2020;6(49):eabd6370.

13. Pei S, Morone F, Liljeros F, Makse H, Shaman JL. Inference and control of the nosocomial transmission of methicillin-resistant Staphylococcus aureus. *Elife*. 2018;7:e40977.

14. Khader K, Thomas A, Huskins WC, et al. Effectiveness of contact precautions to prevent transmission of methicillin-resistant Staphylococcus aureus and vancomycin-resistant enterococci in intensive care units. *Clinical Infectious Diseases*. 2021;72(Supplement_1):S42-S49.
